# Using Monte-Carlo simulation to test predictions about the time-course of semantic and lexical access in reading

**DOI:** 10.1371/journal.pone.0296874

**Published:** 2024-04-02

**Authors:** Conrad Perry

**Affiliations:** Faculty of Health and Medical Sciences, School of Psychology, The University of Adelaide, Adelaide, Australia; University of Glasgow, UNITED KINGDOM

## Abstract

One of the main theoretical distinctions between reading models is how and when they predict semantic processing occurs. Some models assume semantic activation occurs after word-form is retrieved. Other models assume there is no-word form, and that what people think of as word-form is actually just semantics. These models thus predict semantic effects should occur early in reading. Results showing words with inconsistent spelling-sound correspondences are faster to read aloud if they are imageable/concrete compared to if they are abstract have been used as evidence supporting this prediction, although null-effects have also been reported. To investigate this, I used Monte-Carlo simulation to create a large set of simulated experiments from RTs taken from different databases. The results showed significant main effects of concreteness and spelling-sound consistency, as well as age-of-acquisition, a variable that can potentially confound the results. Alternatively, simulations showing a significant interaction between spelling-sound consistency and concreteness did not occur above chance, even without controlling for age-of-acquisition. These results support models that use lexical form. In addition, they suggest significant interactions from previous experiments may have occurred due to idiosyncratic items affecting the results and random noise causing the occasional statistical error.

## Introduction

**Fig 1:**
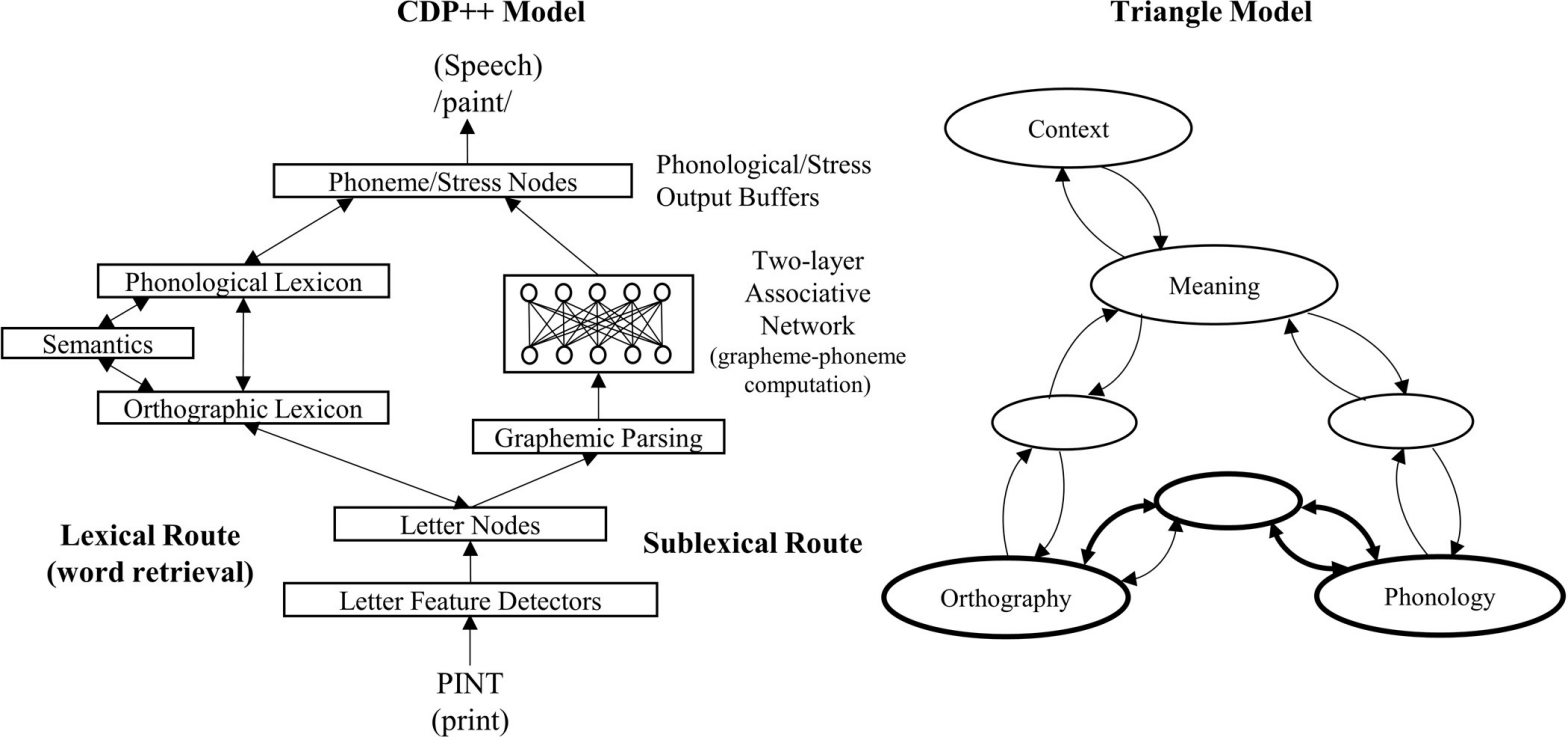
The CDP++ model (left) and the Triangle model (right).

Models of reading differ considerably with respect to how they predict people read. One of the key differences between them is whether there are word-form lexicons for what people think of as orthographic (written) and phonological (spoken) words. Dual-route models such as the Connectionist-Dual Process Model (CDP) [1–6] have these, whereas backpropagation style models such as the Triangle model [7–10] do not (see Fig 1). This means the Triangle model has direct mappings between letters and semantics and thus predicts semantics is accessed very early when reading and that interactions between semantics and phonology should occur. Alternatively, CDP can only predict interactions that occur late in processing [11] (i.e., after lexical form has been accessed).

Two of the most commonly used experimental tasks used to look at semantic and other effects in reading are the reading aloud [12–15] (also called *naming*) and lexical decision task [16–18]. These tasks differ significantly in terms of the size of the spelling-sound consistency effect (c.f., e.g., *pint* vs. *mint*, *tint*, *lint*) they typically produce, which is an indicator of phonological processing. This effect has been greater in naming compared to lexical decision tasks. In lexical decision tasks, null-effects have commonly been reported with normal adult readers (see Stone et al. [19] for an in-depth discussion of why this may be the case), unlike reading aloud where an effect is typically found. As will be seen below, however, spelling-sound consistency effects can be found in the lexical decision task when more modern stimuli selection methods are used, even with normal adult readers in standard experimental conditions.

One way that the use of semantic information when reading has been quantified has been to examine the extent to which spelling-sound consistency and semantic variables interact [20, 21]. Whilst this is not able to definitively show whether semantics is processed early or late [11, 22], interactions are suggestive of a prominent role for semantics in early reading processes, at least when naming tasks are used. The interactions have also been important in helping understand acquired surface dyslexia [23, 24].

A number of studies have examined the extent to which semantics affects RTs in normal reading by examining whether imageable words are faster to read than abstract words. The first studies examining this did not find any evidence for the effect of imageability on reading [25–29]. However, Strain et al. [20] and then later Woollams [30] did, with larger effects of spelling-sound consistency being found with low frequency abstract compared to low frequency imageable words. Further research in this area criticized some of these results based on the characteristics of the items used [29]. For example, the significance of a result in the Strain et al. study was entirely reliant on a single item and the data of Woollams et al. did not reach significance using a by-items test which, at the time, was usually considered the ‘gold-standard’ for reporting significance with the type of analysis they used. Further testing of this and similar effects has suggested that the weak results may be due to individual differences [21, 31], although even some of those results have not necessarily supported initial theoretical claims [21].

One of the reasons mixed results may have been found is simply due to power. Because of the nature of these studies, it is likely to be easy to unknowingly choose biased stimuli sets. This is because RTs in the types of task used to examine such processing do not form a normal distribution, and there is a long right tail [32, 33]. This means individual items may bias the results easily because relatively small numbers of stimuli are used in these experiments (20 items per cell is common), and very slow items are not typically removed from the data analyses–rather, items are typically only removed due to high error rates. Thus, the results of Strain et al. [34] where one item changed the pattern of significance in a critical result should not be seen as highly unlikely since in that study only 12 items per cell were used in one experiment and 16 in the other two. The obvious problem caused because of this is that if a small number of items happen to be very slow in one category (like inconsistent abstract words) compared to another category (like inconsistent imageable words), interactions not representative of the actual distribution may occur.

One likely reason the number of words used in these experiment has often been small is because the number of usable words given database availability until relatively recently was not large enough for adequate stimuli selection. This is because the largest consistency effects have typically been found with low frequency words that are highly inconsistent except under very special conditions[12, 14]. This means that from a full database of words, the number that are usable is reduced greatly because most words are not highly inconsistent and of low frequency. This pool is reduced further because words at either end of the imageability/consistency spectrum need to be chosen. To see how serious this problem is, if words are selected from the monosyllabic database of Spieler and Balota which has 2998 words using cutoff values of 4 or more out of 5 for concreteness (see below for why concreteness was used and not imageability), 0.3 or less for spelling-sound consistency, and 100 or less for frequency (using CELEX counts), there are only 18 usable words.

One way around this problem would be to examine these effects using large-scale regression analyses. Yap and Balota [35] did this using the WordNet senses and semantic density as their semantic variables. They did this with over 6000 words from the English Lexicon Project (ELP) [36]. After controlling for many other variables they found that their semantic variables accounted for .5% and .1% of the variability in lexical decision and naming RTs, where words with many semantic senses and greater semantic density had faster RTs than those with few. In the error data, words with many senses caused more errors, but only in the naming task. These results suggest the effect of semantics, a least in this database, is weak. They also examined the consistency by frequency by semantics interaction. They did not find a significant interaction. A similar non-significant interaction has also been found in a monosyllabic database where imageability was used as the semantic measure [37].

Cortese and Schock [38] performed a similar analyses as Yap and Balota [35], looking at the effect of spelling-sound consistency and imageability with 1936 words also taken from the ELP. In the 10^th^ step of a stepwise regression where numerous other variables were controlled (phonological onset, stress, word length, word frequency, orthographic and phonological neighbourhood, quadratic length, orthographic distance variables, spelling-sound consistency variables, imageability and age of acquisition), no significant interaction was found, although there was a marginal interaction in the error data in the LDT task. However, they found this null effect after controlling for Age of Acquisition (AOA), which has been argued to be a potential confound that could cause non-significant results [30, 31, 39], and thus some groups may not consider the null effect evidence against the effect existing.

Whilst the database analyses of Yap and Balota [35] and Cortese and Schock [38] provided evidence against findings of such an interaction, there are a number of aspects of their studies that could have increased their chance of finding a null effect. There have also been further reports of the interaction being found in small-scale experiments [21, 31]. With the Yap and Balota analyses, the way semantics was measured is quite different to studies where the interaction has been found when either imageability [21, 31] or concreteness [40] has been used, and thus whether the Triangle model would even predict an interaction with those variables is unclear. Alternatively, Cortese and Schock [38] did use imageability. However, the number of words in the database Cortese and Schock used was relatively small, meaning there were probably rather few words at the more extreme end of the distribution (i.e., very low frequency and highly inconsistent) that have been commonly used in experimental studies. This may be problematic because if the interaction is largely found with such words, the regression may not have had the power to capture the interaction even if it exists. Linear regression may also not have been appropriate if the effect is nonlinear and largely occurs at the end of the distribution, which is what is typically found with the consistency by frequency interaction, including in Yap and Balota. The distribution of RTs in naming tasks is also heteroscedastic, with the lower frequency words showing more variability and this may affect the reliability of linear regression [41].

Even ignoring these potential problems, if there is no need to understand the shape of the entire distribution, then finding the interaction may be easier if items are restricted to those likely to show a large effect, which is what has been implicitly done in small-scale experiments where very low frequency items with extreme manipulations have been chosen. It has also been done with databases examining similar measures. For example, when Reilly and Desai [42] examined semantic density using the ELP database after choosing a set of words that were not intercorrelated on other factors, they found a strong effect of it on RTs, unlike when Yap and Balota [35] did not do this.

There are also theoretical arguments that have made interpreting the results of these studies difficult. One is that there are intercorrelations between semantic and other variables and there is no strong agreement on how these should be interpreted. The most important variable in this respect is Age-of-Aquisition (AOA) (see Chang et al. [43] for a summary of this variable and modelling of it). This variable refers to when in life a word is learnt. This variable complicates studies to do with semantics because children tend to learn imageable/concrete words earlier than abstract words. This not only means that a word used at a similar frequency by adults may have a different cumulative across-time exposure frequency, but there are potentially representational differences that could occur based on AOA that affect reading [43]. Some versions of this hypothesis claim that aspects of AOA and semantics are largely or at least partially the same thing [30, 31, 39], and therefore AOA should not be used as a covariate in studies looking at semantics because it could cause and may have been responsible for null-effects that have been found. In this case, the idea is that AOA does not simply make the interpretation of other semantic variables difficult but using it as a covariate removes some of the shared semantic content, hence reducing the amount of ‘semantics’ in any semantic manipulation and potentially causing null effects to be found. In the regression analyses of Cortese and Schock [38], this was not considered as they entered both semantic and AOA measures before examining the interactions.

A second and much less serious problem for this analyses is that semantics is obviously a multidimensional and complicated area, whereas none of the main reading models include any large-scale modelling of semantics as found in the semantics literature [44, 45]. Because of this, whilst most of the studies described above used imageability as a semantic measure, concreteness has also been used, including by some of the proponents of the Triangle model [40] who have also used imageability. Whilst it has long been argued that there are differences between these two constructs [46], the correlation between them is high, and this has been found in many languages [47–49]. In the simulations below, concreteness was used and not imageability because the database [50] was larger than extant imageability databases [51, 52] and thus allows a greater number of words to be used. One might argue that concreteness and imageability would cause different results, and perhaps an interaction could be found using an imageability and not concreteness manipulation. However, if the two constructs elicited different results it would mean that the relatively small amount of non-shared variance that was not due to noise would need to be the source of it. If that could be found, it would be extremely interesting because it would mean that a small part of what imageability is that is not shared with concreteness would be responsible for all of the differences in RTs, and thus represent a very important aspect of what imageability is and concreteness is not. However, that would presumably not be predicted by the Triangle model because the ‘semantics’ it uses has always been very generally defined, typically as simple semantic features (e.g., Harm & Seidenberg [53]).

One way of examining questions to do with semantic involvement in reading is via the use of Monte Carlo simulation [54, 55]. Monte Carlo simulation can be done on extant data, of which there is now a large amount in the area of reading. It can also be used to create sets of experiments similar to actual experiments and estimates of their power can be derived. Given the debates to do with semantics, here, I will investigate the extent to which three factors (age-of-acquisition, spelling-sound consistency, and concreteness) affect reading in both lexical decision and naming tasks. Using Monte-Carlo simulation is especially useful because it is possible to avoid the problem of intercorrelated variables found in regression analyses. This can be done by selecting small groups of items where variables that are often intercorrelated are matched across groups on many variables. This means that, compared to regression analyses, problems to do with assumptions and how to interpret covariates is reduced. In addition, because more recent databases that are available contain many more usable words (i.e., they have more words and associated information about them such as frequency of usage) than were historically available, it is also possible to test the extent to which results may differ when a greater diversity of words is used. In this case, the preponderance of effects examined only using English monosyllables, which was historically common, and the effect it may have had can be elucidated. Thus, it is possible to explore different effects in theoretically different and interesting conditions. Finally, with Monte-Carlo simulation, the actual distribution of results of pseudo-experiments from different item sets can be generated [54, 55]. This distribution is simple to visualize so the likely power of an experiment using randomly chosen words can be easily ascertained.

Monte-Carlo simulation is not without problems when run to simulate experiments. Notably, if matched words need to be sampled based on multiple criteria, then some words may be sampled more commonly than other words simply because they tend to be easier to match than other words. Thus, like actual small-scale experiments, the simulations may not be perfectly representative of all words. In addition, with these simulations, because large numbers of different experiments need to be created, large numbers of words and their associated characteristics are needed. Without them, the amount of times the same words that are used across different experiments would increase, causing the samples to be less independent, although there are ways of examining if this is actually a problem [55].

There are a number of issues that will be investigated here. First, since AOA is theoretically interesting, a number of comparisons will be run where this variable is and is not controlled for in other comparisons. Notably, both spelling-sound consistency, concreteness, and interactions thereof will be examined with and without controlling for AOA. This will give insight into the extent to which AOA actually affects results on critical manipulations. Since it has been common to look for effects with only low frequency words in many studies in this area, results from frequency restricted vs. non-restricted databases will also be compared where possible. Monosyllabic databases will also be compared to databases with additional words. Even in cases where it is not possible to select enough words due to not enough words existing for a particular manipulation when the databases are restricted in some way, this is useful to know. In this case, it means the set of possible words is so restricted that studies in the area are likely to use overlapping words and hence idiosyncratic item properties if they exist would be difficult to avoid. Third, it is possible to examine different effects from the same population to get some idea of how they and confounding variables might affect results when more complex designs are used. Thus, it is simple to run multiple pseudo-experiments without having subject variability across experiments to deal with. In terms of the experiments that were run, for the sake of simplicity, initially pairwise comparisons were used between all of the main variables of interest (frequency, concreteness, AOA, and spelling-sound consistency). Because of the theoretical importance of the spelling-sound consistency by concreteness interaction, this was also run as a 2 × 2 ANOVA with items matched across quadruplets rather than pairs.

## Simulations

### Method

#### Initial data processing

The monosyllabic databases used in Perry et al. [55] were all used here. These included the naming RTs for 2806 monosyllabic words from Spieler and Balota [56] (both young and old participants—which will be referred to as the Spieler Young and Spieler Old databases) and Seidenberg and Waters [8], the monosyllabic naming and lexical decision RTs from Balota et al. [36], and the monosyllabic lexical decision RTs from Keuleers et al. [57] (note that a small number of words are missing in some of the databases, so not all databases had exactly 2806 monosyllables). Expanded versions of the Balota et al. [36] naming/lexical decision databases and the Keuleers et al. database that included disyllables were also used. These databases all had over 20,000 words.

Lexico-statistics for the monosyllabic words were created from stimuli using information from CELEX [58], apart from concreteness and AOA. This meant the statistics were consistent across all the words and simulations that I have previously run elsewhere [55] and in numerous simulations with reading models [1–3, 59]. They are also from the database from which stimuli from many older experiments were chosen from, as for a long time it was the largest and most comprehensive database people used. The lexico-statistics used were: word frequency, orthographic neighbourhood, spelling-sound consistency (using body/rime units and count frequencies), concreteness [50], and age of acquisition [60]. Lexico-statistics (frequency and orthographic neighbourhood) for the expanded Balota et al. [61] (lexical decision and naming) and Keuleers et al. [57] databases were taken from the ELP [61], as the words from the ELP are better overlapped with those databases because the statistics taken were derived directly from the Balota Naming/Lexical Decision data. This included using the HAL frequency counts. This means the lexico-statistics can differ slightly when the same word is used with different databases (i.e., the monosyllabic and expanded databases). Statistics for the expanded databases were not calculated for spelling-sound consistency in the ELP and so were taken from Perry et al. [2] where those statistics were calculated for all monosyllabic and disyllabic words. When words were disyllabic, the lowest spelling-sound consistency score from the two possible syllables was used.

#### Picking matched stimuli

**Fig 2:**
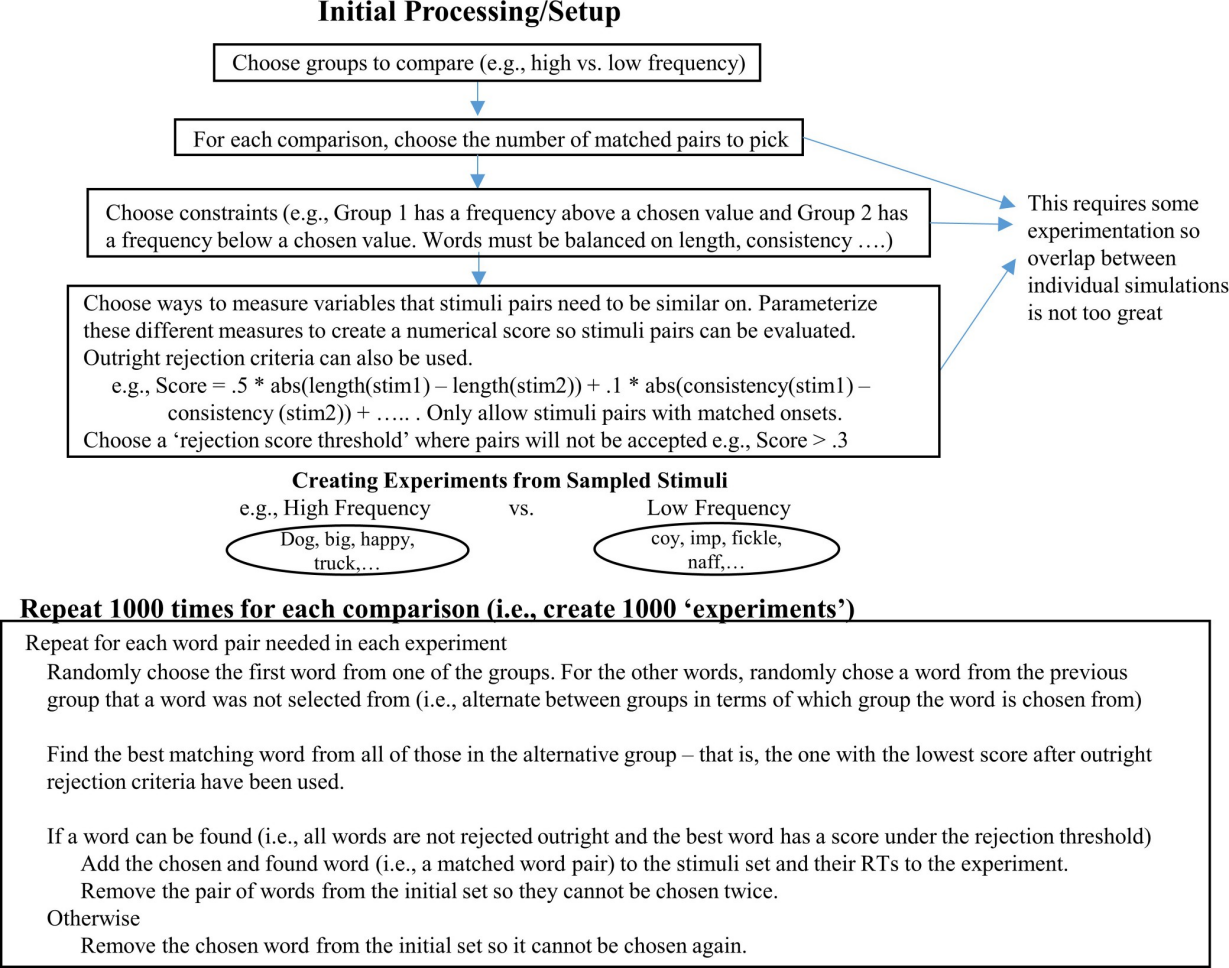
Summary of the way the words are chosen in the different experiments.

To generate ‘experiments’ for the different comparisons, items need to be picked for them. This was done in a very similar way as Perry [55] where pairs of matched items were chosen until enough were found for each experiment (see Fig 2 and a more verbose description in the S1 File).

The following procedure was used:

1. Word pairs were selected by first choosing a word (the ‘target word’) from one of the groups in a comparison at random and then pairing it with the word in the alternative group that it had the best match with (the ‘matching word’). For example, in a word frequency manipulation, the minimum high frequency and maximum low frequency values would be chosen. The target word would then be chosen from one or other of those groups (e.g., a high frequency word). The matching word would then be found from the other group (i.e., the low frequency words).
2. To choose the matching word, all words in the matching group were scored on a matching criterion, and the word with the lowest (best) value was chosen as the match.
3. The matching criteria were specified differently depending on the contrast, but the criteria could include word frequency, orthographic neighbourhood, word length, onset phoneme, spelling-sound consistency, and concreteness. Some of these criteria could cause an outright rejection. These included the initial phoneme of words not matching in the naming databases, word length being different, and the number of orthographic neighbors differing too much. In many cases, there were words that did not have a value from one of the criteria used for matching because it did not exist in one of the databases used (e.g., no concreteness value). When this was the case, the word was considered unmatchable and not used. All of the values used appear in the S1 Data.
4. If no outright rejection criteria were triggered, a quantitative score for each word below the threshold that needed to be matched was calculated. The score was simply the sum of the absolute distances between the target word and matching word calculated from the different criteria. For example, if log frequency was used as a matching criteria, part of the score would include |log frequency (target)–log frequency (matching)|.
5. A parameter was chosen by hand for each of the different matching criteria so that the difference between the variables that were supposed to be matched on all of those criteria was minimized. Further simulations run that are not reported here showed that varying these parameters within reasonable bounds did not make much difference to the simulations (i.e., bounds that meant that the mean value of a variable did not differ meaningfully across the two groups when it was not supposed to). Different parameters are needed because the absolute difference between scores can differ a lot. For example, a target and matching word might have a difference of 10 when examining orthographic neighbourhood. This is greater than the difference between the least frequent and most frequenct word based on log word frequency. Thus, if parameters were not used to reduce the scoring for neighborhhood, it would simply dominate the selection criteria at the expense of other variables and the groups would end up confounded on other variables.
6. Once the best match was found, it was checked against a maximum discrepancy score to see whether it really was a reasonable match. This was done because it is possible to choose a target word from the database that matches no other word well.
7. Once the pair had been selected, their RTs were saved for later analyses and the words were removed from the database from which they were selected so they could not be used twice. If a word had no reasonable match, it was removed from the database.
8. The next pair of words was chosen but instead of first choosing a target word from the category that was previously used, it was chosen from the other category. For example, if a word frequency manipulation was being examined, a word pair would first be chosen where the target word was above a high frequency threshold and the matched word below a low frequency threshold. The next pair chosen would then use a target word with a frequency below the low frequency threshold and a word with a frequency above the high frequency threshold would then be matched to it.
9. This continued until enough pairs had been found per group, thus creating an experiment where there were two groups of RTs that were pairwise matched.
10. This entire process was repeated until enough simulations for each comparison were generated.

For the ANOVAs, the procedure was essentially the same as choosing pairs of items. However, instead of choosing the word from which the others were then matched and going back and forward between the two groups, a word was chosen and then the other three words were matched to it. The group that each new word was chosen from was cycled through the 4 groups. The matching score for each of the sets of 4 words chosen was taken from the worst match of the three words that were searched for. If this was above the given rejection criteria, the 4 words were not used, and the initial target word that was chosen was removed from the database so it could not be chosen again. Given the importance of this comparison, different simulations were done with multiple frequency cut-offs. This was done by first finding a value where the parameters used meant that the groups only averaged a maximum of around 20% overlap. In this case, it meant only items with a log 10 frequency less than 3.5 were used (a raw count frequency of 3163 occurrences–as a comparison, the highest frequency word ‘the’ occurs around 23 million times). Additional simulations using a log frequency less than 3.0 (1000 occurrences), 2.5 (317 occurrences), and 2.0 (100 occurrences) were also run. These simulations give a more extreme manipulation, but more shared items are used across the individual simulations, so their results need to be taken with caution as the same words are potentially more likely to be used in many sets. Error rates were also examined the same way. For the sake of simplicity, only the results of the RTs of the log(3.5) frequency group are reported below in full details unless specifically noted (see the S2 Data for the full results).

The number of word pairs used was 30 for all of the experiments that used the small databases. For the experiments using the three expanded databases, initially the results were examined using 100 matched pairs. For a small number of comparisons, it was not possible to generate reasonable sets of words without very high overlap between sets from these values. For the words selected from the small databases, these were not run. With the larger databases, the number of matched pairs or matched groups of 4 for the ANOVA analyses were reduced so that each experiment that was simulated did not use, on average, more than 22.5% of the same items as the other simulated experiments in any of the groups. The average number of items that were overlapped in each simulation with all other simulations in each comparison was also calculated for each of the two possible groups in the paired test or four possible groups in the ANOVAs. This was done by examining the number of overlapped items in all possible simulation pairings. For example, if only 3 simulations per comparison were used, overlap would be calculated for the 1^st^/2nd, 1^st^/3rd, and 2^nd^/3rd pairings. With 100 simulations, this generates 4951 pairings. This gives a proportion overlap score. For example, .33 means that with any given simulation, on average, 33% of the same items in the same group as the other simulations in that comparison would be overlapped.

Once all of the experiments were created, with the simple experiments, a simple paired t-test was used on each one to determine if there was a significant difference between the two groups of items that they were created from. Outliers were removed by calculating the difference in RTs for each word pair and removing any pair that had a difference that was 3SDs above or below the mean difference. With the ANOVAs, a repeated measures design was also used. The initial simulations were done without removing outliers, although further simulations were also done to examine the effect of outliers and assumptions of ANOVA. These were done to strengthen the validity of the results.

### Results

All simulations for each of the comparisons were run 1000 times. Using a chi-squared distribution, this means that if 150 significant *p*-values are found that go the same direction (e.g., high frequency words are faster than low frequency words) then it suggests that the significant *p*-values occurred more often than chance. This will be referred to as a ‘significant’ difference and will be used to refer to the overall comparison. When the number of significant *p*-values in each experiment is reported, this will be noted as such. The results appear in Table 1 and S1 Fig in S1 File. Differences between groups on all of the measures examined can also be found in the S1 Data.

**Table 1:** Number of significant simulations and median differences (ms) across the different comparisons. Note: two *p*-values are used to represent each end of the distribution. The two possible significant conditions are given in brackets below the main comparison (e.g., low frequency words being faster than high frequency words, and high frequency words being faster than low frequency words). Var1 and Var2 are mean scores of the variables that were manipulated in the comparison. When ‘significance’ is stated in the summaries it means overall significance (i.e., 150+ significant individual results).

Comparison (Direction of effect)	Database	Overlap G1	Overlap G2	N. pairs	N. p < .05	N. p < .05	Median RT dif (ms)	Var 1	Var 2
**Log 10 Frequency**	Spieler_Young_N	0.19	0.24	30	0	820	14.50	3.54	1.13
(Low<High, High<Low)	Spieler_Old_N	0.19	0.23	30	0	989	37.47	3.55	1.14
	Seid_Waters_N	0.19	0.23	30	0	715	26.18	3.54	1.14
	Balota_N (Mono)	0.18	0.23	30	0	955	43.95	3.54	1.13
	Balota_LDT (Mono)	0.18	0.24	30	0	1000	127.08	3.54	1.13
	Keuleers_LDT (Mono)	0.18	0.24	30	0	1000	113.90	3.54	1.11
	Balota_N (All)	0.16	0.07	100	0	1000	87.46	3.71	1.07
	Balota_LDT (All)	0.17	0.08	100	0	1000	140.54	3.71	1.07
	Keuleers_LDT (All)	0.18	0.08	100	0	1000	125.42	3.71	1.12
Summary	High frequency word are consistently faster than low frequency words.								
**Concreteness**	Spieler_Young_N	0.11	0.06	30	10	51	2.18	2.06	4.55
(Abs < Conc, Conc < Abs)	Spieler_Old_N	0.11	0.06	30	2	145	8.13	2.06	4.55
	Seid_Waters_N	0.11	0.06	30	43	10	-1.27	2.07	4.55
	Balota_N (Mono)	0.10	0.05	30	8	64	5.31	2.07	4.55
	Balota_LDT (Mono)	0.10	0.05	30	1	406	27.12	2.07	4.55
	Keuleers_LDT (Mono)	0.10	0.05	30	1	590	24.91	2.07	4.55
	Balota_N (All)	0.02	0.01	30	14	67	3.05	1.92	4.53
	Balota_LDT (All)	0.02	0.01	30	1	188	17.60	1.92	4.53
	Keuleers_LDT (All)	0.02	0.01	30	1	318	20.09	1.92	4.53
Summary	Concrete words tend to be faster than abstract words in LDT databases, but the effects are fairly weak.								
**Concreteness (Low Frequency)**	Spieler_Young_N	0.21	0.10	30	9	29	1.97	2.10	4.53
(Abs < Conc, Conc < Abs)	Spieler_Old_N	0.21	0.09	30	0	116	8.07	2.10	4.52
	Seid_Waters_N	0.21	0.09	30	56	14	-2.86	2.11	4.53
	Balota_N (Mono)	0.20	0.09	30	6	49	1.63	2.11	4.53
	Balota_LDT (Mono)	0.20	0.09	30	0	356	27.18	2.11	4.52
	Keuleers_LDT (Mono)	0.20	0.09	30	0	632	27.46	2.11	4.53
	Balota_N (All)	0.21	0.12	70	60	2	-4.84	1.95	4.50
	Balota_LDT (All)	0.22	0.12	70	15	22	0.55	1.95	4.51
	Keuleers_LDT (All)	0.22	0.12	70	0	261	13.32	1.95	4.50
Summary	Concrete words tend to be faster than abstract words in LDT databases, but the effects are fairly weak.								
**AOA**	Spieler_Young_N	0.10	0.12	30	410	0	-8.13	5.16	10.69
(Early < Late, Late < Early)	Spieler_Old_N	0.10	0.12	30	455	0	-16.55	5.16	10.69
	Seid_Waters_N	0.10	0.12	30	130	1	-10.01	5.15	10.70
	Balota_N (Mono)	0.09	0.12	30	268	0	-16.67	5.13	10.66
	Balota_LDT (Mono)	0.09	0.12	30	947	0	-56.11	5.11	10.66
	Keuleers_LDT (Mono)	0.09	0.12	30	995	0	-52.69	5.11	10.70
	Balota_N (All)	0.10	0.08	100	983	0	-32.02	5.31	11.02
	Balota_LDT (All)	0.10	0.08	100	1000	0	-55.49	5.30	11.01
	Keuleers_LDT (All)	0.10	0.08	100	1000	0	-51.66	5.31	11.01
Summary	Early AOA words are faster than late AOA words, with strong effects found in the LDT databases and the full Balota Naming database.								
**AOA** **(Low Frequency)**	Spieler_Young_N	0.14	0.09	30	283	0	-6.93	5.30	10.81
(Early < Late, Late < Early)	Spieler_Old_N	0.15	0.09	30	469	0	-17.62	5.30	10.82
	Seid_Waters_N	0.14	0.09	30	64	6	-6.08	5.31	10.80
	Balota_N (Mono)	0.13	0.09	30	246	1	-17.16	5.26	10.79
	Balota_LDT (Mono)	0.14	0.09	30	941	0	-56.66	5.26	10.79
	Keuleers_LDT (Mono)	0.13	0.08	30	987	0	-51.47	5.26	10.86
	Balota_N (All)	0.24	0.07	30	612	0	-41.41	5.57	11.57
	Balota_LDT (All)	0.23	0.07	30	589	0	-44.19	5.58	11.57
	Keuleers_LDT (All)	0.25	0.08	30	917	0	-55.16	5.60	11.56
Summary	Early AOA words are faster than late AOA words but the effects differ a lot in power across the databases.								
**Concreteness (Low Frequency, AOA Matched)**	Spieler_Young_N	0.27	0.12	30	19	10	0.55	2.12	4.47
	Spieler_Old_N	0.27	0.12	30	19	23	0.52	2.12	4.47
(Abs < Conc, Conc < Abs)	Seid_Waters_N	0.26	0.11	30	62	11	-5.14	2.12	4.47
	Balota_N (Mono)	0.25	0.11	30	28	15	-1.64	2.12	4.47
	Balota_LDT (Mono)	0.25	0.11	30	3	72	12.09	2.12	4.47
	Keuleers_LDT (Mono)	0.26	0.11	30	4	95	9.37	2.12	4.47
	Balota_N (All)	0.40	0.21	100	569	0	-20.45	1.95	4.50
	Balota_LDT (All)	0.41	0.21	100	82	0	-8.56	1.95	4.50
	Keuleers_LDT (All)	0.41	0.22	100	9	21	-0.15	1.95	4.50
Summary	The only significant effect was in the full Balota Naming database where abstract words were faster than concrete words.								
**AOA** **(Low Frequency, Concreteness Matched)**	Spieler_Young_N	0.14	0.10	30	201	1	-5.80	5.31	10.63
(Early < Late, Late < Early)	Spieler_Old_N	0.14	0.10	30	365	0	-15.20	5.32	10.62
	Seid_Waters_N	0.13	0.10	30	43	6	-4.20	5.32	10.62
	Balota_N (Mono)	0.13	0.09	30	166	0	-14.09	5.27	10.65
	Balota_LDT (Mono)	0.13	0.09	30	900	0	-49.71	5.28	10.66
	Keuleers_LDT (Mono)	0.13	0.09	30	974	0	-44.85	5.27	10.66
	Balota_N (All)	0.73	0.26	100	989	0	-32.80	5.61	11.26
	Balota_LDT (All)	0.73	0.26	100	966	0	-33.97	5.61	11.25
	Keuleers_LDT (All)	0.78	0.28	100	1000	0	-49.05	5.64	11.28
Summary	Early AOA words are faster than late AOA words, with strong effects found in the LDT databases and the full Balota Naming database.								
**Consistency**	Spieler_Young_N	0.19	0.06	30	1	129	4.28	0.96	0.23
(Inc < Cons, Cons < Inc)	Spieler_Old_N	0.19	0.06	30	0	314	12.63	0.97	0.23
	Seid_Waters_N	0.18	0.06	30	1	174	12.72	0.96	0.23
	Balota_N (Mono)	0.19	0.06	30	0	264	16.44	0.96	0.23
	Balota_LDT (Mono)	0.18	0.06	30	6	55	9.41	0.96	0.23
	Keuleers_LDT (Mono)	0.18	0.06	30	4	83	6.68	0.96	0.23
	Balota_N (All)	0.03	0.02	100	0	784	23.13	0.96	0.19
	Balota_LDT (All)	0.03	0.03	100	0	581	20.52	0.95	0.19
	Keuleers_LDT (All)	0.03	0.03	100	0	463	14.48	0.95	0.19
Summary	Consistent words tend to be faster than inconsistent words, although the strength of the effect differs considerably across databases.								
**Consistency (LF)**	Spieler_Young_N	0.38	0.11	30	0	109	4.85	0.96	0.22
(Inc < Cons, Cons < Inc)	Spieler_Old_N	0.38	0.11	30	0	573	19.62	0.96	0.22
	Seid_Waters_N	0.37	0.11	30	1	225	15.06	0.96	0.23
	Balota_N (Mono)	0.38	0.11	30	0	577	26.35	0.96	0.23
	Balota_LDT (Mono)	0.38	0.11	30	11	24	6.98	0.96	0.23
	Keuleers_LDT (Mono)	0.36	0.11	30	0	72	8.84	0.96	0.22
	Balota_N (All)	0.08	0.08	100	0	949	37.46	0.95	0.19
	Balota_LDT (All)	0.09	0.08	100	0	424	19.75	0.95	0.19
	Keuleers_LDT (All)	0.09	0.08	100	0	328	14.60	0.95	0.19
Summary	Consistent words tend to be faster than inconsistent words, although the strength of the effect differs considerably across databases								
**Consistency ‐ Concrete Only, Low Frequency**	Balota_N (All)	0.18	0.23	40	0	335	21.94	0.93	0.20
(Inc < Cons, Cons < Inc)	Balota_LDT (All)	0.18	0.23	40	0	392	26.34	0.93	0.20
(AOA controlled)	Keuleers_LDT (All)	0.19	0.23	40	1	143	14.17	0.93	0.20
Summary	Consistent concrete words tend to be faster than inconsistent concrete words, although the strength of the effect is weak.								
**Consistency ‐ Abstract Only, Low Frequency**	Balota_N (All)	0.16	0.13	50	0	659	27.62	0.95	0.18
(Inc < Cons, Cons < Inc)	Balota_LDT (All)	0.16	0.13	50	0	245	19.22	0.95	0.19
(AOA controlled)	Keuleers_LDT (All)	0.17	0.14	50	3	66	7.82	0.95	0.18
Summary	At least in the Balota databases, consistent words are faster than inconsistent words, with a stronger effect found in the naming database.								
**Consistency–Concrete Only, Low Frequency**	Balota_N (All)	0.14	0.15	40	0	572	30.73	0.94	0.20
(Inc < Cons, Cons < Inc)	Balota_LDT (All)	0.14	0.16	40	0	394	26.88	0.94	0.20
(not pairwise AOA matched)	Keuleers_LDT (All)	0.14	0.16	40	0	281	19.16	0.94	0.20
Summary	Consistent concrete words are faster than inconsistent concrete words with weak-medium effects found across the databases.								
**Consistency–Abstract Only, Low Frequency**	Balota_N (All)	0.15	0.12	50	0	600	27.62	0.95	0.18
(Inc < Cons, Cons < Inc)	Balota_LDT (All)	0.16	0.12	50	0	328	22.27	0.95	0.18
(not pairwise AOA matched)	Keuleers_LDT (All)	0.16	0.13	50	3	69	8.92	0.95	0.18
Summary	At least in the Balota databases, consistent words are faster than inconsistent words, with a stronger effect found in the naming database.								

Note: Spieler_Young_N = Spieler & Balota (young participants, naming), Spieler_OId_N = Spieler & Balota (old participants), Seid_Waters_N = Seidenberg & Waters (naming) Balota_N = Balota et al. (naming), Balota_LDT (lexicial decision task), Keuleers_LDT = Keuleers et al. (lexical decision task), Mono = only monosyllabic words, All = full database, Median dif = difference in medians, LF = Low frequency items only, Abs = Abstract, Conc = concrete, Inc = inconsistent, Conc = Consistent. Log Frequency: Var 1 = log 10 (High Frequency), Var 2 = log 10 (low Frequency).

Initial inspection of the results showed that it was not possible to examine consistency with only concrete or abstract words of low frequency with the monosyllabic databases without stimuli overlapping excessively across experiments–that is, each word set generated would have shared, on average, much more than 22.5% of its words with the other item sets, a level I took as too high to maintain the validity of the results. Therefore, these comparisons were only run with the expanded databases. Similarly, stimuli used to examine the effect of concreteness controlled for AOA as well as spelling-sound consistency using low frequency words with the monosyllabic databases had a relatively high overlap, although the results are left in but need to be interpreted with caution.

#### Comparisons

*Word frequency*. The results showed that that the effect of word frequency was strong across all databases, with results from the expanded databases being somewhat greater than the monosyllabic ones.

*Concreteness (full set of items) and concreteness (only low frequency words)*. There were similar effects of concreteness in both the full and frequency restricted databases. Significant effects were only found with the lexical decision databases, and even these had relatively low power. Notably, only the Keuleers monosyllabic database had significant p-values occurring more than 50% of the time.

*AOA (full set of items) and AOA (only low frequency words)*. The effect of AOA of was significant in all but two comparison ‐ the Seidenberg and Waters naming database was not significant with the full or frequency restricted items.

*Concreteness (only low frequency words with stimuli controlled for AOA)*. There were no comparisons that reached significance apart from with the expanded Balota naming database. With that database, the results showed that abstract words were read faster than concrete ones, the opposite of the most commonly reported pattern. These results suggest that AOA is an important confound and that it significantly reduces the effect of concreteness when it is controlled.

*AOA (only low frequency words with concreteness controlled)*. All of the comparisons reached significance, except the Seidenberg and Waters database. This suggests that effects of AOA are unlikely to be largely due to a confound with concreteness.

*Spelling-sound consistency (full set of items and only low frequency words)*. With the expanded databases, there were significant effects found in all groups apart from the monosyllabic lexical decision databases and the Spieler Young database. With the frequency restricted databases, stronger effects of spelling-sound consistency were found, suggesting low frequency words produce a greater spelling-sound consistency effect. This is consistent with most studies apart from when stimuli with very specific characteristics have been examined [14]. The fact that effects of spelling-sound consistency were found in the expanded lexical decision databases but not the databases with only monosyllabic words is interesting. In this case, if only monosyllabic databases were run, it may have been reasonable to stick with the common assumption that lexical decision tasks do not show or only show very weak consistency effects. However, strong versions of this assumption (i.e., that there is no effect) are clearly incorrect as shown by the results from the expanded databases. Note that the significant effects of spelling-sound consistency in the expanded lexical decision databases did not occur simply because there were a greater number of stimuli in the simulations than the monosyllabic databases. The average size of the RT difference in the expanded databases was also greater than in the monosyllabic databases.

A second interesting aspect of the results was that it was not possible to generate a lot of word sets without overlapping stimuli with only low-frequency words in the monosyllabic databases. This shows that there are simply not enough monosyllabic/monomorphemic words to create a diversity of different experiments without reusing the same words. Thus, whilst the experiments in this area generally produce the same sort of results (i.e., a significant spelling-sound consistency effect), if there are words with idiosyncratic effects, then it is likely they would get commonly reused across studies. That is, some items could have properties that affect their reaction times (RTs) that cannot be controlled for by using standard lexicostatistics, and this is not just random noise (see e.g., Forster [62], for a discussion). This could potentially provide a somewhat biased view of the strength of the spelling-sound consistency effect if a pool of items that are commonly used across experiments had these properties and they tended to go one direction (i.e., cause RTs either higher or lower than the lexicostatistics would suggest).

*Spelling-sound consistency (Low frequency words only*: *Concrete and abstract controlled for AOA*. *Stimuli taken from only the expanded databases)*. Both the abstract and concrete words produced similar results where inconsistent words were slower to read than consistent ones. Significant results were found in both the naming and lexical decision databases of Balota, unlike the Keuleers database. With the Balota naming database, there were more significant *p*-values with the abstract than concrete words (34% vs. 66%). However, the number of items used with the concrete words was less than used with the abstract words due to differences in the number of usable stimuli (40 vs. 50 per group). The overall median difference in the size of the spelling-sound consistency effect with the Balota naming database was only 6 ms, with the abstract words showing a greater effect. The opposite pattern was found with the lexical decision databases, where a greater median difference was found with the concrete compared to abstract words, although it was small (7ms and 6ms for the Balota and Keuleers databases, respectively).

*Spelling-sound consistency (Low frequency words only*: *Concrete and abstract words not controlled for AOA)*. The simulations produced relatively similar results to the same simulations where items were pairwise matched on AOA except for a greater number of significant *p*-values, especially with the Keuleers lexical decision database with concrete words. The concrete words also showed greater consistency effects than the abstract words with the median effect being 3ms, 5ms, and 10ms greater for concrete versus abstract words for the Balota naming, Balota lexical decision, and Keuleers database, respectively.

#### Spelling-sound consistency by concreteness ANOVA (Low frequency words only, controlled for AOA and not controlled for AOA)

**Fig 3:**
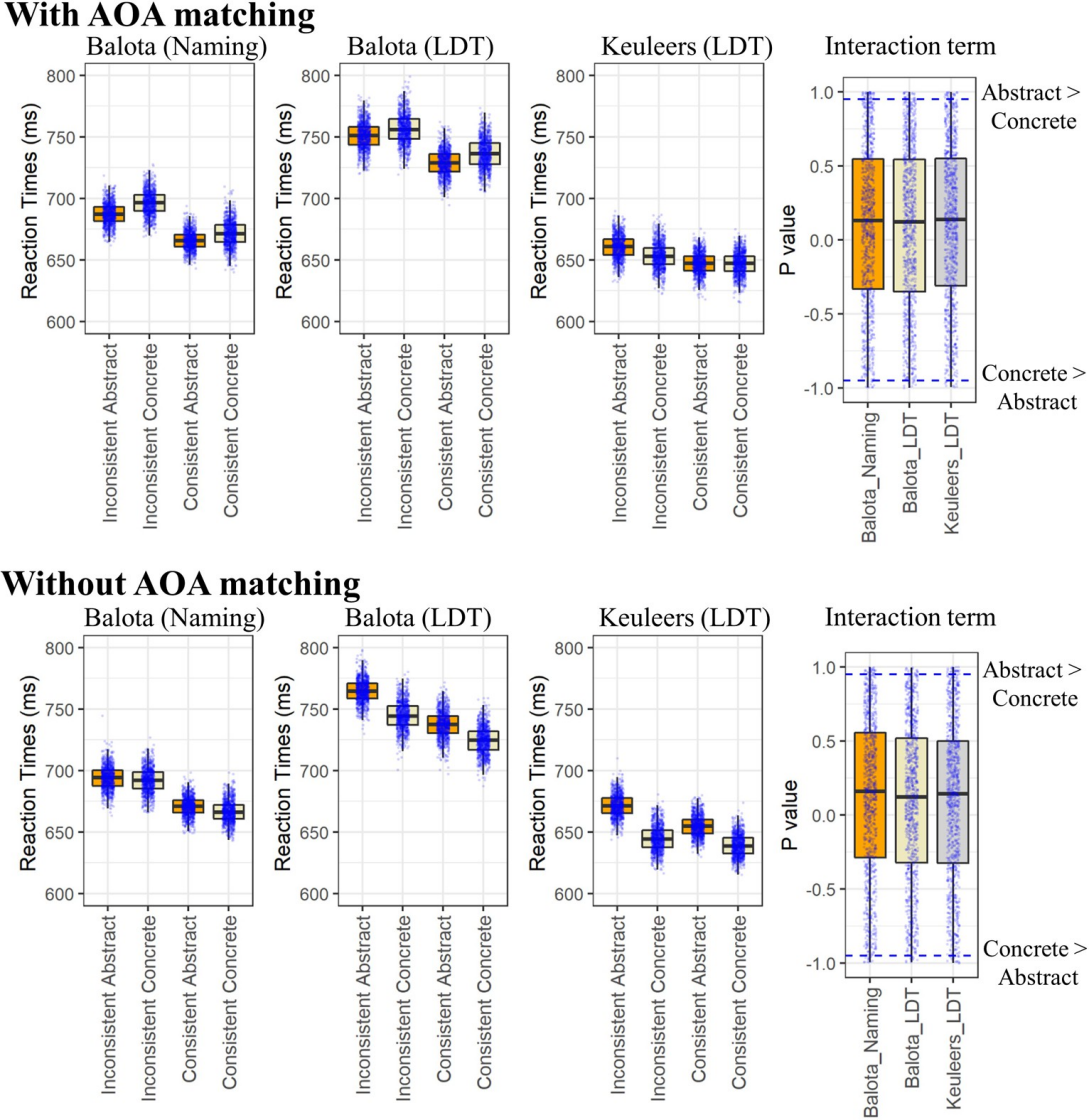
RTs and flipped-p values from the simulations of the Spelling-sound Consistency by Concreteness ANOVA for the simulations with AOA matched stimuli (top) and without AOA matched stimuli (bottom). The blue dots are results from the individual simulations. With the interaction term, positive values on the Y axis represent one minus the p value (1 –p). Thus, the closer to 1 the smaller the p value. The negative values represent represent–(1 –p). Thus, the closer to -1 the smaller the p value. The dots above and below the dotted blue line are significant at p < .05. The whiskers in both panels represent 1.5 +/- the interquartile range or the maximum/minimum value in the graph. Note: LDT = Lexical Decision.

**Fig 4:**
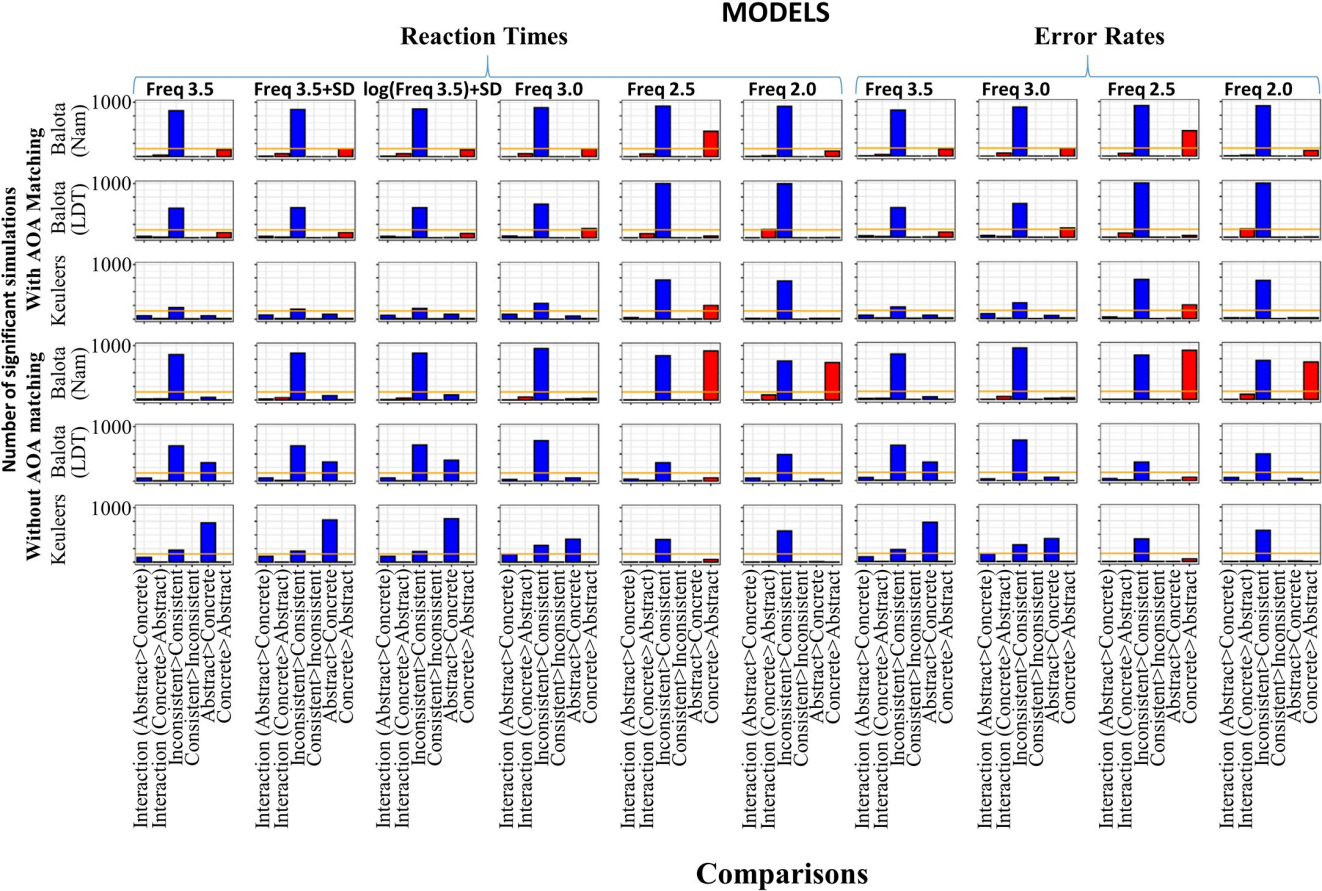
Number of significant results from 1000 simulations that produced a significant concreteness × consistency interaction or main effect of consistency or concreteness as a function of response type (reaction times and error rates), database used, and which model generated the results. The orange line is the criterion for overall significance (i.e., 150 individual significant simulations).

The median difference in the Balota naming dataset was 3.8ms, with concrete words showing a greater consistency effect than abstract words when AOA was matched, and 1.7ms when it was not. With the LDT databases the consistency effect showed the opposite pattern where the abstract words showed a greater consistency effect than concrete words. The difference in the simulations that controlled for AOA were 2.4ms and 7.8ms, for the Balota and Keuleers database, respectively, and 6.9ms and 9.9ms for the same databases when AOA was not controlled. There were not enough significant individual *p*-values from any of the comparisons for the interactions to reach the overall significance threshold in the RTs. Alternatively, there was a significant difference with the Keuleers database in the error rates, with the abstract words showing a greater consistency effect than concrete words (3.50%; 188 significant simulations). There were also significant main effects of consistency across all of the databases, although they were small in the Keuleers database (10ms and 12ms in the matched and non-matched sets, respectively). Alternatively, main effects of concreteness were only found in the Balota LDT and Keuleers databases when AOA was not matched. The error results were somewhat similar to the RTs in that if something was significant in the RTs it was also likely to be significant in the errors. However, there were many cases where there was a significant effect on RTs but not errors. The results from the simulations can be seen in Fig 3 and the number of significant results found in terms of RTs and errors rates for all of the models examined can be seen in Fig 4. Full data on all measures appears in the S1 Data for this and the rest of the simulations.

Even though ANOVA with the same number of items in each cell is robust to assumption violations [63], two further simulations were done to test the assumptions of the main results. These were done because when the RTs were examined, significant normality violations using a Kolmogorov-Smirnov test even with a relatively low significance level (*p* < .01) were found. In one of the simulations, quadruplets were removed when any one of the RTs in the four groups was above or below a 3SD threshold calculated from each group separately. In the other simulation, log RTs were used and outliers were also removed. The results showed that the SD cut-off did not remove excessive numbers of outliers (.27% in the raw RT and .11% in the log RT simulations–note that this removes four times the number of items due to the removal of quadruplets when one item is an outlier). The log RTs also produced much more normal distributions (see S2 Fig in S1 File). However, in terms of the pattern of data and the number of significant results found, there were few differences. This suggests that the ANOVAs using raw data were robust to potential statistical problems due to assumption violations. The results of the significance values found in the RT and error data in these and all of the other simulations run can be seen in Fig 4. Given these two new models produced essentially the same results as the original model with the log(3.5) frequency, they will not be discussed further. Note that given the distribution of the error rates (most items had very low error rates and only a few had high error rates), I did not try to transform them so they were normally distributed since comparing results when the entire shape of the distribution is changed would not have been especially meaningful.

The results suggest that, in the naming task, matching words on AOA or simply allowing them to be confounded when words are only matched on concreteness does not make much difference to the interaction results. In terms of the other results, all of the databases showed a significant overall consistency effect, although the results were weak in the Keuleers database. Concreteness effects were also found in the two LDT databases, although only when the stimuli were not matched on AOA.

Apart from the results from the group with highest frequency cut-off, a significant interaction did emerge in RTs with the errors in the Keuleers database using the log(3.0) frequency group. Those results displayed a 5.0% effect where the abstract words showed a greater consistency effect than the concrete words, with 198 individual simulations reaching significance. These results are somewhat surprising because the Keuleers database showed the weakest consistency effect and the task was lexical decision. This suggests that the difference may not have been caused by the effect of spelling-sound consistency on semantics but rather due to idiosyncratic properties of the words.

One reason potentially idiosyncratic differences may have affected the lower frequency results is that the overlap between the same stimuli used in different experiments was greater (see Table 2). Notably, the average overlap in the Balota naming database for the log (3.0), log (2.5), and log (2.0) groups was 21.9%, 37.5%, 70.8%, with a maximum overlap for any condition of 29.6%, 54.1%, and 90.7%. The maximum overlap condition always occurred with the consistent abstract words. These results show that when very low frequency words are used, the word pool is very limited even with very large databases. Interestingly, whilst the results did not reach significance even with the reduced number of items, the consistency effect with concrete words was still numerically greater than the consistency effect with abstract words in the Balota naming database. Across the three lower frequency groups in the Balota naming database the effect was 7.0ms, 8.3ms, and 21.8ms with the AOA matched words and 7.0ms, 2.1ms, and 25.6ms with the AOA unmatched words for the log (3.0), log (2.5), and log (2.0) groups, respectively.

**Table 2:** Mean proportion overlap of words in the four different sets of experiments.

Manipulation	Database	Simulation Frequency Cutoff															
		Log 3.5 (Main simulations)				Log 3.0				Log 2.5				Log 2.0			
		Inc Abs	Inc Conc	Cons Abs	Cons Conc	Inc Abs	Inc Conc	Cons Abs	Cons Conc	Inc Abs	Inc Conc	Cons Abs	Cons Conc	Inc Abs	Inc Conc	Cons Abs	Cons Conc
AOA Matched	Balota Naming	.21	.13	.22	.14	.29	.19	.30	.20	.52	.37	.54	.33	.91	.74	.89	.66
	Balota LDT	.21	.12	.19	.13	.27	.17	.23	.18	.43	.29	.39	.28	.78	.60	.72	.52
	Keuleers	.21	.12	.19	.13	.27	.18	.24	.19	.43	.30	.40	.29	.81	.63	.75	.55
AOA Unmatched	Balota Naming	.20	.13	.22	.13	.28	.18	.28	.19	.51	.34	.52	.29	.89	.70	.87	.59
	Balota LDT	.19	.12	.19	.11	.25	.16	.24	.16	.42	.28	.40	.26	.78	.59	.75	.51
	Keuleers	.19	.12	.19	.11	.25	.17	.24	.16	.42	.30	.40	.27	.80	.63	.76	.56

Note: Inc = inconsistent, Cons = consistent, Abs = abstract, Conc = concrete

Apart from how the frequency cutoffs affected the results, another factor that could have been important is the database used. This is because there are arguments about which database has the best frequency counts [64] and word frequency had a strong effect on the data. Given this, I investigated whether the results may have be driven by the particular database used by switching the word frequency counts from HAL to SUBTLEX. The simulations using a log (3.5) frequency cutoff were then rerun. The results were very similar, although interestingly the overlap between experiments was reduced from an average of 16.3% with a maximum of 22.3% for the most overlapped condition (which was the consistent abstract words) using the HAL frequency counts to an average of 12.4% with a maximum of 16.6% for the most overlapped condition (which was also the consistent abstract words) using the SUBTLEX frequency counts. This suggests that the results were not driven by word frequency counts specific to one database. The results appear in the S2 Data. I also examined the initial pairwise comparisons with SUBTLEX rather than CELEX frequencies. The results were very similar, although the SUBTLEX frequencies tended to produce more significant results. The raw results appear in the S1 Data.

## General discussion

In this study, I used Monte Carlo simulations across a number of lexical decision and naming databases to examine a number of factors thought to be important in reading aloud and lexical decision tasks, notably AOA, concreteness, and spelling-sound consistency. There were a number of somewhat unexpected results to do with spelling-sound consistency and concreteness found across the different databases. Results from different versions of the same database were also interesting and showed how important it is to use more than just monosyllabic stimuli in studies.

One of the surprising results was that the two expanded lexical decision databases showed significant effects of spelling-sound consistency. This was not an especially strong result (58.1% and 46.3% of the *p*-values in the simulations showed a significant difference in the expanded Balota and Keuleers databases using all of the words and 42.4% and 32.8% of the *p*-values were significant with only low frequency words), but is contrary to the most common finding with normal adult readers where no significant effect is reported. When the same word set was used except restricted to monosyllables, none of the comparisons reached significance. These results show that spelling-sound consistency effects do occur in lexical decision tasks, even under conditions where nonword fillers are not especially difficult [19]. Apart from being interesting in its own right, this result meant that examining spelling-sound consistency and how it interacts with concreteness was worth doing, even with lexical decision tasks.

In terms of the concreteness effect, whilst the results showed that concrete words tended to be faster to read than abstract words, at least in lexical decision tasks, this was only when AOA was not matched across groups. When pairwise matching of AOA was used with the stimuli, the results showed that there were no significant differences across the databases apart from the expanded Balota naming database where abstract words had significantly faster RTs than the concrete words in 56.9% of the simulations. Interestingly, in a regression analyses Cortese and Khanna [65] performed that also used the Balota et al. database, no significant effect of imageability was found in the naming data when AOA was controlled. Finding that abstract words were faster than concrete words after controlling for AOA is surprising but not unheard of, although why it only occurred in one of the databases is not clear. Barber et al. [66], for example, has also reported such a find from a lexical decision task. Barber et al. also noted that this was not the typical find and referenced three other studies that showed concrete words were faster than abstract ones [67–69]. Inspection of those studies [67–69] showed that none controlled for AOA. Whilst examining large numbers of papers on concreteness and seeing what was done with AOA is out the scope of this article, it is clear even more recent papers examining concreteness often do not consider AOA at all (e.g., Perry et al. [70]). Whilst semantic and AOA effects may well be related, this means that effects that are likely to originate from non-semantic aspects of AOA [39] may potentially be a confound in some studies.

Apart from concreteness being examined by itself, the interaction between spelling-sound consistency and concreteness also showed few significant effects when AOA was matched across groups. The results were fairly straight forward. With the comparisons where concrete and abstract words were examined separately, a consistency effect was found in both groups. This was when AOA was controlled and when it was not controlled. Importantly, the size of the spelling-sound consistency effect was also very similar with both concrete and abstract words.

Given the results of the pairwise comparisons where spelling-sound consistency was examined with abstract and concrete words separately, the results of the ANOVAs examining the same thing but with stimuli matched across all groups, including AOA, were unsurprising. The results also showed that the size of the difference in the spelling-sound consistency effect with concrete and abstract words was very small.

Apart from the number of *p*-values not reaching overall significance in the ANOVAs, the results from the individual simulations also give some insight into why different results may have been reported. As can be seen from Fig 3, the individual simulations showed that a small number of simulations were significant where abstract words showed a greater spelling-sound consistency effect than concrete words and vice-versa. Given this and the distribution of results, one would expect that the occasional stimuli set that was seemingly reasonably matched would still produce a significant interaction.

The results from the simulations with AOA controlled were also interesting. Notably, they showed that failing to control for AOA did not much have much of an effect on the size of the interaction. With the full Balota naming database, the difference between the size of the spelling-sound consistency effect with abstract and concrete words was 3.8 ms and in the AOA controlled database it was 2.8ms. The main effects did change slightly, with the spelling-sound consistency effect increasing a small amount and the size of the concreteness effect increasing in the lexical decision databases. Thus, whilst AOA clearly affects the results, it does not appear to be an important confound that would otherwise make interpreting the interaction difficult. The small size of the difference across the two conditions is perhaps not surprising. This is because even when concreteness was manipulated and the stimuli were not controlled for AOA, the actual differences in AOA were relatively small. The abstract inconsistent words had an average AOA of 2.46 more years than the concrete inconsistent words and the abstract consistent words had an average AOA of 2.03 more years than the concrete consistent words. A similar pattern was found in the results of Woollams et al. [21]. Thus, even though AOA produces a relatively strong effect by itself when extreme manipulations are used to examine it, the size of the manipulation when AOA was not controlled was relatively small and this is likely to be responsible for the weak effect it had. This suggests that arguments about which covariates should be used and the extent to which semantics is overlapped with AOA do not actually matter much for this particular question.

### Could individual differences invalidate these results?

An important issue that has not been considered that is relevant to more recent work is the idea that individual differences may be important in understanding the interaction between spelling-sound consistency and concreteness. At least with the main effect of spelling-sound consistency, the standard result that is almost always found is that slow readers produce greater consistency effects than fast readers (see Yap et al. [71] for an in-depth discussion). More recent studies have also found that the speed at which people read (and other measures of reading ability) is positively correlated with the difference between the size of the spelling-sound consistency effect people show with abstract compared to concrete words [31, 40]. That is, it has been argued that abstract words show a greater effect of spelling-sound consistency effect concrete words, but that this effect is largely caused by slow readers. Such a result may explain why this pattern did not occur in the simulations reported here if there were no slow readers in the databases.

Despite not having the data for individuals apart from the Balota database, it is possible to get some idea of the extent to which differences existed across the databases that were used. This is because the databases differed considerably in terms of how fast the average reader was and how large the spelling-sound consistency effect was. Notably, the Spieler Young, Seidenberg and Waters, and Balota naming (monosyllabic) databases were largely equivalent in terms of the actual words in the databases. However, the size of the spelling-sound consistency effect with low frequency words differed substantially, with the median consistency effect found across the simulations being 5.63ms, 15.16ms, and 24.59ms, and the number of significant differences found in the simulations being 9%, 19%, and 61%, for the Spieler Young, Seidenberg and Waters, and Balota naming (monosyllabic) databases, respectively. The median overall RTs also went in the same direction, being 477ms, 582ms, and 647ms, for the same three databases. This shows that, at least at the group level, the slower the readers were, the greater the spelling-sound consistency effects they showed, just as one might expect. That is, the spelling-sound consistency effect in the Balota naming database was much greater than the other databases, and the overall RTs in the Balota naming database were also much slower. The effect size with the expanded Balota naming database was even greater, with the spelling-sound consistency effect being 35.85ms.

Given that there were large differences in the size of the spelling-sound consistency effect in the different naming databases, with the Balota et al. database showing the greatest spelling-sound consistency effect, this suggests there are likely to have been more slow readers in that database than the other ones. Thus, if it is true that individual differences are responsible for the interaction, then one would predict a stronger interaction in the Balota database than the other databases because there must have simply been more readers who displayed a larger spelling-sound consistency effect. However, as noted previously, this group displayed essentially no difference between the size of spelling-sound consistency effect with abstract and concrete words, and thus this seems very unlikely.

Whilst the results from the Balota et al. naming database suggest a difference compared to other studies, it does not offer a reason as to why other experiments have shown a correlation between the difference in the size of the spelling-sound consistency effect between abstract and concrete words and reader speed. One possibility is that rather than think about the individual results as a difference in the spelling-sound consistency effect, they can be thought of as a slow-gets-slower effect. That is, if a word is difficult to process, the difference between the speed at which it is processed compared to words that are easier to process will tend to be greater in readers who are slower at reading in general. Such a pattern exists in other psycholinguistic variables [72] such as nonword length and frequency. This means if a stimuli set is confounded to start with, such as when inconsistent abstract words are used that are slower to name than inconsistent concrete words, then the effect of the confounds will be likely to be greater in slow compared to fast readers. Thus, a significant correlation between the difference in size of the spelling-sound consistency effect between abstract and concrete words and reader speed would simply represent a stimuli set that was initially confounded and not something to do with underlying processes driving a difference between abstract and concrete words.

Whilst the difference between databases may offer a useful explanation of some of the results, the results need to be taken with caution. One reason for this is that the experimental set-ups across the different studies were different. Thus, some of the differences could have come from things like the microphone responding differently or how participants interpreted the instructions given, although a 170ms overall difference caused by microphone differences would be very surprising. A second difference between studies is how many stimuli the participants read. In the Balota et al. database, the participants only read a subset of the words that was less than the earlier studies. Thus, participants may have been more willing to respond quickly in the Balota et al. database than the others. This, if anything, predicts responses should have been faster in that database compared to the other ones. A less obvious reason is that the differences across databases could have come from the homogenization of response latencies (see Lupker et al. [73]) where people’s RTs are to some extent homogenized based on the variability of the stimuli they respond to due to articulatory processes. This may have affected the size of effects caused by reading processes across the databases because there was less variability in the monosyllabic compared to disyllabic databases. This may have reduced the extent to which homogenization occurred in the Balota naming database compared to the others and thus caused the responses to be comparatively slower than the other databases.

If the individual results from some of the other databases were to become available, it would be useful to examine the proportion of participants who showed a consistency effect and compare them with the Balota et al. database. This is because not all participants show a consistency effect in naming studies, and it tends to be slower subjects who show the larger effects (see Yap et al. [71] for a thorough discussion). Thus, if the participant groups were otherwise similar and the differences were caused by homogenization, this would predict a similar proportion of participants would show a consistency effect across the databases but the effect size would be greatest in the Balota et al. database. Alternatively, if the difference was due to more vs. less skilled participants, it would predict that more participants would show a consistency effect in the Balota et al. database compared to the other databases.

### General implications

The results have import theoretical implications. Notably, they suggest that significant interactions between consistency and semantic variables reported in previous studies may have been due to idiosyncratic stimuli or statistical anomalies. These results are consistent with those found in the database analyses of Cortese and Schock [38] who showed that in standard regression analyses, no significant interaction was found.

The simulations using lower frequency words showed how such idiosyncratic effects could emerge. In this case, even though the RT results were not significant, the size of the interaction increased as lower frequency words were used. In this respect, with the lowest frequency word sample, the consistency effect with concrete words was 22ms and 20ms greater than the consistency effect with the abstract words in the matched and unmatched AOA groups, respectively—the results from the main simulation were 3.8ms and 1.7ms with the same comparisons. The reverse pattern was found with the error rates, where a significant interaction was found in the reading aloud data where inconsistent abstract words had a higher error rate than inconsistent concrete words in the unmatched AOA simulations in the lowest frequency group. However, the median difference in the error rate of the interaction was only 1.3%, with the abstract words having the higher error rate. This suggests that words coming from this very restricted set (77% of the words were overlapped, on average, across the experiments) could cause idiosyncratic results. This is an important observation because it sheds light on historical findings. This is because it shows that the limited number of usable words could increase the likelihood of an effect being driven by idiosyncratic word properties and these words would often be shared across experiments. This could potentially lead to an effect being found across many experiments due to the same potentially idiosyncratic items being used.

Monte-Carlo simulations extend such results and are useful because they allowed multiple item sets to be tested and power estimates to be gained in a similar way to running actual experiments. The way stimuli are chosen was also repeatable and transparent, typically unlike stimuli chosen by hand. In addition, because stimuli can be balanced across variables rather than regressed out, difficulties with statistical assumptions to do with regression were avoided at the price of having to run many different experiments. Finally, as discussed above, it allowed a number of hypotheses to be tested including whether the effect only occurred in low frequency words and the extent to which confounds with AOA affects the results.

Apart from the lack of significant findings in the main data set, the distribution of the effects showed that the interaction had low power and was not producing reliable results. This suggests that if the difference between the consistency effect with abstract and inconsistent concrete words was correlated with other tasks such as reading efficiency, interpreting the results would be difficult. This is because the low power suggests the results have poor reliability. Therefore, if correlations were found, they may not be reflective of the size of the difference in the consistency effect between abstract and concrete words, but rather other correlated factors that these variables may reflect. As discussed above, such correlations may be more likely to represent a slow-get-slower effect.

## Conclusion

Overall, the results here show that Monte Carlo simulation can provide great insight into commonly debated effects. Notably, by initially examining simple effects that could contribute to semantic access in reading and potentially confound the results, it was possible to get a good understanding of how confounding variables affect the data. Importantly, AOA, which has been implicated as a variable likely to effect the strength of the consistency effect with abstract and concrete words, actually had very little effect. This was despite AOA being a much stronger effect than concreteness. These results are complementary with large-scale regression analyses [38], and allowed stronger hypothesis testing on lower frequency parts of the distribution that may have been important. Together, these two different methods suggest that significant interactions are not found when all words are used in a regression and are also not found when only low frequency words are used that might have been more be likely to show such an interaction. The simulations also allowed power estimations to be gathered for items similar to that used in experiments where many psycholinguistic variables are held constant but the effect of consistency and concreteness examined. They showed that there was no obvious trend towards a significant interaction where abstract words show a bigger consistency effect than consistent ones. The simulations thus provide strong evidence against the predictions of the Triangle model of reading that abstract words with inconsistent spelling-sound mappings access semantics earlier than concrete words with inconsistent spelling-sound mappings and also help understand why previous results have been found.

## Supporting information

S1 Data (XLSX)

S2 Data (XLSX)

S1 File (DOCX)
