# Supplementary material for: Using Monte-Carlo simulation to test predictions about the time-course of semantic and lexical access in reading
Source: PLoS One. 2024 Apr 2;19(4):e0296874. doi: 10.1371/journal.pone.0296874 (PMC10986942; doi:10.1371/journal.pone.0296874)
Supplement: S1 File — (DOCX) [file pone.0296874.s003.docx]

Supplementary Materials

**How words are selected**

The basic idea of the procedure is to try to mimic the way people choose stimuli by hand. The first thing that needs to be done is to determine what the manipulation is. For example, for a word frequency manipulation examining low and high frequency words, values need to be chosen for what low and high frequency values actually are. These values need to be chosen so that they form an appropriate manipulation but also chosen so there are enough items in the two different groups so that many experiments can be created with words sets that do not overlap too much.

Once the values have been chosen, the actual words for simulating individual experiments need to be chosen. This was done in matched pairs or quadruplets so that each individual word in one category was matched with words in the other category (i.e., a low frequency word was chosen that was as close as possible to a high frequency word on different psycholinguistic variables apart from word frequency).

Since it is possible to consider many variables at the same time (often unlike choosing stimuli by hand) a method of determining when a word is an acceptable match needs to be chosen. To do this, a word is first selected from one of the categories, and the entire database is then searched for the word which is the best match for it.

The best match is determined by selecting a group of criteria that measures how well a pair is matched and then creating a score from it. First outright rejection criteria are used. For the frequency contrast, the word being searched for had to be of an equal letter length to the chosen word, the number of orthographic neighbors that differed had to be less than 5, and the difference in consistency had to be less than .2. These values were chosen by hand. If none of these outright rejection criteria were triggered, a score was created. In this case:

Score = abs(log(Frequency Word 1) – log(Frequency Word 2)) / 1 + abs(Consistency Word 1 – Consistency Word 2) / 1 + abs(Neighborhood Word 1 – Neighborhood Word 2) / 20.

As can be seen, the difference in neighbourhood score is divided by 20. This is done because the scores that it produces are large compared to log frequency and consistency. This stops it from dominating the total score. If it was not done, the score for word pairs with similar numbers of orthographic neighbors but large frequency and consistency differences would be lower than words with similar frequency and consistency scores but a greater difference in neighbours. When the parameters are picked within reasonable bounds, the differences in scores will tend to be minimized on all of the measures. There are also outright rejection criteria that can be used, such as matched pairs having to be an equal letter length.

Once all possible word pairs in the pool have been evaluated as a match, the one with the lowest (best) score is chosen. The word is then allowed as a match if it is under a score that suggests that it is an acceptable match. The score for an acceptable match was 1.5 in the pairwise comparisons, and this was chosen by hand. After this, if the match was acceptable, then both the chosen and the searched for word are removed from the selection pool. If the match was not acceptable, then only the chosen word was removed from the selection pool.

This processes was repeated 99 times more so there were 100 word pairs chosen (this number differed depending on the number of stimuli used in the experiment), and these were considered one experiment. After this all of the words were put back into the selection pool. The process was then repeated 999 times more so there were 1000 experiments.

*Figure S1*. Top Panel: Flipped-p values from the simulations using simple contrasts. Positive values on the Y axis represent one minus the ‘flipped’ *p* value (1 – *p*). Thus, the closer to 1 the smaller the *p* value. Negative values represent –(1 – *p*). Thus, the closer to -1 the smaller the *p* value. The blue dots are results from the individual simulations. The dots above and below the dotted blue line are significant at *p* < .05. Bottom Panel: RT differences from the simulations using simple contrasts (ms). The whiskers in both panels represent 1.5 +/- the interquartile range or the maximum/minimum value in the graph. Note: conc = concrete, abs = abstract, Nam = naming, LDT = Lexical Decision, SY = Spieler Young, SO = Spieler Old, SW = Seidenberg and Waters, BN = Balota baming (monosyllabic), BL = Balota lexical decision (monosyllabic), KM = Keuleers (monosyllabic), BN_A = Balota naming all, BL_A = Balota lexical decision (all), K_A = Keuleers (all).

Normality Checks

Apart from the main simulations, as described in the manuscript, a number of further simulations were run to test assumptions of the ANOVA. These were done because it is well known that RT data is not typically normally distributed (e.g., Andrews & Heathcote, 2021), and item outliers based on reaction times are not typically removed from the data analyses. Therefore, all four groups in the ANOVA were subject to a Kolmogorov-Smirnov test, and a count of the number that cause *p* values less than < .01 was made from the 1000 simulations. As can be seen, the models often produced violations of the normality assumption, although this was far less common when the RT data was log transformed. Removing outliers also helped. The number of assumption violations for the error rates are not displayed as almost all of the distributions were non-normal as is typical of the naming and lexical decision tasks (i.e., most items show relatively few errors, with a very long tail). The individual results for all of the simulations can be found in the spreadsheet in the spreadsheet in the Supplementary Materials.

**S2 Fig**. Number of results out of the 1000 simulations that produced a significant normality violation based on a Kolmogorov-Smirnov test (p < .01) as a function of database used and which model generated the results. The orange line is the criterion for significance (150 significant simulations).
